# Research advances in the study of Campylobacter, Helicobacter, and Related Organisms

**DOI:** 10.3389/fcimb.2012.00159

**Published:** 2012-12-19

**Authors:** D. Scott Merrell, Alain Stintzi

**Affiliations:** ^1^Department of Microbiology and Immunology, Uniformed Services UniversityBethesda, MD, USA; ^2^Department of Biochemistry, Microbiology and Immunology, University of OttawaOttawa, ON, Canada

Campylobacter spp. and Helicobacter spp. are gastrointestinal pathogens that remain a major cause of acute gastroenteritis and gastric disease, respectively. The 16th International Workshop on Campylobacter, Helicobacter and Related Organisms (CHRO) was organized by Erin Gaynor and Christine Szymanski and was held in Vancouver, BC, Canada from August 28 to September 1, 2011. This meeting highlighted recent advances in our understanding of the epidemiology, survival mechanisms, host response, and pathogenesis of these important species. This Research Topic issue highlights each of these topics and attempts to shed insight into our growing understanding of the process of host-pathogen interactions as it relates to Campylobacter and Helicobacter. We wish to dedicate this Research Topic to Dr. Andre Dubois, who passed away unexpectedly on June 30, 2012. We are honored that one of his research articles appears in the issue and note that his loss is deeply felt by our community.

A substantive overview of the CHRO meeting is provided by Drs. Gaynor and Szymanski (2012) and the articles within this Research Topic are broadly divided into those dealing with Campylobacter and then Helicobacter. Those articles related to Helicobacter can broadly be divided into three major topics: those that address *H. pylori* lifestyle and biological processes, those that address *H. pylori* virulence factors, and those that address interaction of *H. pylori* with the host.

Within the *H. pylori* lifestyle and biological processes, studies from the Dubois lab investigate the mechanism by which *H. pylori* enters host cells (Liu et al., 2012). Though primarily considered an extracellular pathogen, it is clear that a fraction of *H. pylori* cells enter and survive within the host cell. The Dubois team presents evidence that NudA is important in this process. Next, Liechti and Goldberg review the process of membrane biogenesis in *H. pylori* and compare it to the processes that have been elucidated in *Escherichia coli* and *Neisseria meningitidis*; not surprisingly, *H. pylori* often does not follow the paradigms established in these model systems (Liechti and Goldberg, 2012). Finally, Pernitzsch and Sharma discuss transcriptome complexity and riboregulation in *H. pylori* (Pernitzsch and Sharma, 2012). It has only recently become evident that *H. pylori* employs post-transcriptional regulation and small regulatory RNAs (sRNAs) as a mechanism of gene regulation. This fact directly conflicts the previous dogma that life within the singular niche of the host stomach has led to *H. pylori*'s loss of complex gene regulation.

Among *H. pylori* virulence factors, CagA, and VacA are undoubtedly the best studied. As such, a number of articles are devoted to these important factors. The Solnick group presents evidence that expression of genes on the *H. pylori cag* pathogenicity island, which encodes for CagA, varies significantly, and that the organization of the genes into transcriptional units is conserved among several *H. pylori* strains (Ta et al., 2012). In terms of CagA delivery, the Backert group investigates sequences/domains within the CagL protein that are important for interaction with integrins and subsequent injection of CagA into host cells (Conradi et al., 2012). Next, the Guillemin group describes the utilization of a novel Drosophila system to identify host components that affect CagA activity within host cells (Reid et al., 2012). Finally, Kim and Blanke review the role of the VacA toxin in modulation of the gastric epithelium and discuss the understudied area of VacA and CagA interaction (Kim and Blanke, 2012).

The final major topic addressed among the *H. pylori* manuscripts is the consequences of *H. pylori*-host cell interaction. From the bacterial perspective, the Cover group specifically discuss what we currently know about how host cell contact alters *H. pylori* cells (Johnson et al., 2012). Conversely, Noto and Peek discuss our current understanding of the role of microRNAs on the process of *H. pylori* pathogenesis and gastric carcinogenesis (Noto and Peek, 2012). Finally, the Müller group tackles an intriguing question that is coming to the forefront of the *H. pylori* field; does colonization with *H. pylori* actually provide any benefits to the host? Recent studies suggest that this is indeed the case, and Müller et al. review how *H. pylori* immunomodulation can confer protection against allergic and chronic inflammatory disorders (Arnold et al., 2012).

Those articles related to Campylobacter can be divided into five major topics: the function of glycans and capsule polysaccharides in Campylobacter virulence and biology, the mechanism of cell invasion, Campylobacter antimicrobial resistance, molecular typing methods, and general Campylobacter metabolism and biology.

Capsular polysaccharides (CPS) protect microbes from environmental insults and host immune defenses. Guerry et al. review our current knowledge on the role of CPS in Campylobacter virulence and provide an interesting perspective on the potential of CPS as a conjugate vaccine (Guerry et al., 2012). In an original research article, Sorensen et al. demonstrate that the *O-methyl* phosphoramidate (MeOPN) moiety of the *C. jejuni* CPS is recognized as a receptor by several different phages (Sorensen et al., 2012). Interestingly, they observed *in vivo* phase variation of the capsular structure leading to phage resistance and suggesting phage-host co-evolution. Day et al. highlight the key role of *C. jejuni* surface glycans in its interaction with the host and the function of the host glycoconjugates in the defense against *C. jejuni* infection (Day et al., 2012).

Epithelial cell invasion is thought to be a major determinant of *C. jejuni* virulence. One review article and two original research papers focus on this important virulence trait (Boehm et al., 2011; Croinin and Backert, 2012; Neal-McKinney and Konkel, 2012). Boehm et al. used knockout cell lines to characterize the host signaling cascades involved in the process of *C. jejuni* invasion (Boehm et al., 2011). This study provides clear evidence of a role for the fibronectin, integrin beta1, FAK, and DOCK180/Tiam-1 signaling cascade and for Rac1 GTPase activation in *C. jejuni* epithelial cell invasion. Neal-McKinney and Konkel demonstrate that the flagellum secreted protein CiaC is delivered into the cytosol of the host cells and interact with the cell signaling pathways involved in *C. jejuni* cell invasion (Neal-McKinney and Konkel, 2012), possibly the signaling cascade identified by Boehm et al.

Colonization of the host gastrointestinal tract is intimately linked to the ability to survive host defenses. In this regard Hoang et al. describe the identification of genetic loci enabling *C. jejuni* to resist fowlicidin-1 exposure, a potent chicken antimicrobial peptide (Hoang et al., 2012). Intriguingly, fluoroquinolone-resistant Campylobacter have been previously shown to exhibit enhanced *in vivo* fitness. In an original research article, Han and collaborators now confirm the role of the Thr-86-Ile mutation in *gyrA* in conferring fluoroquinolone-resistance and demonstrate that this mutation modulates fitness *in vivo* and DNA supercoiling homeostasis in Campylobacter (Han et al., 2012). Given the importance of DNA supercoiling in gene expression, Han and collaborators argue that the altered DNA supercoiling observed in the fluoroquinolone-resistant Campylobacter might be directly linked to its increased fitness. Clearly, the current increase in the prevalence of fluoroquinolone-resistant Campylobacter threatens clinical treatments. This concern prompts Dufour et al. to assess the potential of phytochemicals as preservative to reduce *Campylobacter* load in food (Dufour et al., 2012). This work reports the antimicrobial properties of isothiocyanates (ITC) against 24 isolates of *C. jejuni* demonstrating their bactericidal effects and highlights the role of the γ-glutamyl-transpeptidase gene in Campylobacter resistance to ITC.

The detection of Campylobacter outbreaks is hampered by the lack of defined and standardized methods to unambiguously detect and track sources of Campylobacter. Two research articles tackle this important epidemiological issue. Through the use of publically available whole genome sequences of *C. jejuni* and *C. coli*, Carrillo and collaborators describe the development of a framework to assess the performance of the existing and emerging molecular typing methods (Carrillo et al., 2012). In terms of the multilocus sequence typing methods (MLST), Miller and collaborators describe and assess the performance of four MLST methods for differentiating strains of the emerging Campylobacter species *C. hyointestinalis*, *C. lanienae*, *C. sputorum*, *C. concisus*, and *C. curvus* (Miller et al., 2012).

Finally, one original research article and four reviews highlight the recent developments in our understanding of Campylobacter physiology and biology. Haddad et al. describe the pleiotropic role of the polynucleotide phosphorylase (PNPase) in *C. jejuni* biology which affects motility, cell invasion and adherence, and chick gut colonization (Haddad et al., 2012; Matos et al., 2012). Plummer reviews our current knowledge on quorum-sensing in Campylobacter and its role in pathogenesis (Plummer, 2012). Alemka and collaborators describe Campylobacter physiology at the mucosa-luminal interface and specifically the complex interplay between *C. jejuni* and mucus (Alemka et al., 2012). Stahl et al. summarize Campylobacter ability to acquire and metabolize nutrients including a discussion of L-fucose metabolism in *C. jejuni* (Stahl et al., 2012). And lastly, Kaakoush and Mitchell highlight the emerging role of *Campylobacter concisus* as a new player of acute and chronic intestinal disease (Kaakoush and Mitchell, 2012).

Clearly we have learned a significant amount about Campylobacter and Helicobacter over the course of the last decades. However, as stated by Gaynor and Szymanski “both pioneers and new investigators in the CHRO research field continue to obtain ‘unexpected results’ demonstrating that Campylobacters and Helicobacters do not follow classic paradigms of other well-characterized gastrointestinal pathogens and we are learning that there is a plethora of interesting related organisms beyond *C. jejuni* and *H. pylori*.” Therefore, though we have learnt much, it is clear that the coming years offer many places to advance our understanding of these important pathogens.

## References

[B1] Alemka A. Corcionivoschi N. Bourke B. (2012). Defense and adaptation: the complex inter-relationship between *Campylobacter jejuni* and mucus. Front. Cell. Inf. Microbio. 2:15 10.3389/fcimb.2012.00015 22919607 PMC3417559

[B2] Arnold I. C. Hitzler I. Muller A. (2012). The immunomodulatory properties of *Helicobacter pylori* confer protection against allergic and chronic inflammatory disorders. Front. Cell. Inf. Microbio. 2:10 10.3389/fcimb.2012.00010 22919602 PMC3417532

[B3] Boehm M. Krause-Gruszczynska M. Rohde M. Tegtmeyer N. Takahashi S. Oyarzabal O. A. (2011). Major host factors involved in epithelial cell invasion of *Campylobacter jejuni*: role of fibronectin, integrin beta1, FAK, Tiam-1, and DOCK180 in activating Rho GTPase Rac1. Front. Cell. Inf. Microbio. 1:17 10.3389/fcimb.2011.00017 22919583 PMC3417370

[B4] Carrillo C. D. Kruczkiewicz P. Mutschall S. Tudor A. Clark C. Taboada E. N. (2012). A framework for assessing the concordance of molecular typing methods and the true strain phylogeny of *Campylobacter jejuni* and *C. coli* using draft genome sequence data. Front. Cell. Inf. Microbio. 2:57 10.3389/fcimb.2012.00057 22919648 PMC3417556

[B5] Conradi J. Tegtmeyer N. Wozna M. Wissbrock M. Michalek C. Gagell C. (2012). An RGD helper sequence in CagL of *Helicobacter pylori* assists in interactions with integrins and injection of CagA. Front. Cell. Inf. Microbio. 2:70 10.3389/fcimb.2012.00070 22919661 PMC3417467

[B6] Day C. J. Semchenko E. A. Korolik V. (2012). Glycoconjugates play a key role in *Campylobacter jejuni* infection: interactions between host and pathogen. Front. Cell. Inf. Microbio. 2:9 10.3389/fcimb.2012.00009 22919601 PMC3417407

[B7] Dufour V. Alazzam B. Ermel G. Thepaut M. Rossero A. Tresse O. (2012). Antimicrobial activities of isothiocyanates against *Campylobacter jejuni* isolates. Front. Cell. Inf. Microbio. 2:53 10.3389/fcimb.2012.00053 22919644 PMC3417524

[B8] Gaynor E. C. Szymanski C. M. (2012). The 30(th) anniversary of *Campylobacter*, *Helicobacter*, and related organisms workshops-what have we learned in three decades? Front. Cell. Inf. Microbio. 2:20 10.3389/fcimb.2012.00020 22919612 PMC3417558

[B9] Guerry P. Poly F. Riddle M. Maue A. C. Chen Y. H. Monteiro M. A. (2012). Campylobacter polysaccharide capsules: virulence and vaccines. Front. Cell. Inf. Microbio. 2:7 10.3389/fcimb.2012.00007 22919599 PMC3417588

[B10] Haddad N. Tresse O. Rivoal K. Chevret D. Nonglaton Q. Burns C. M. (2012). Polynucleotide phosphorylase has an impact on cell biology of *Campylobacter jejuni*. Front. Cell. Inf. Microbio. 2:30 10.3389/fcimb.2012.00030 22919622 PMC3417634

[B11] Han J. Wang Y. Sahin O. Shen Z. Guo B. Shen J. (2012). A fluoroquinolone resistance associated mutation in gyrA affects DNA supercoiling in *Campylobacter jejuni*. Front. Cell. Inf. Microbio. 2:21 10.3389/fcimb.2012.00021 22919613 PMC3417464

[B12] Hoang K. V. Wang Y. Lin J. (2012). Identification of genetic loci that contribute to Campylobacter resistance to fowlicidin-1, a chicken host defense peptide. Front. Cell. Inf. Microbio. 21:32 10.3389/fcimb.2012.00032 22919624 PMC3417529

[B13] Johnson E. M. Gaddy J. A. Cover T. L. (2012). Alterations in *Helicobacter pylori* triggered by contact with gastric epithelial cells. Front. Cell. Inf. Microbio. 2:17 10.3389/fcimb.2012.00017 22919609 PMC3417513

[B14] Kaakoush N. O. Mitchell H. M. (2012). *Campylobacter concisus*– a new player in intestinal disease. Front. Cell. Inf. Microbio. 2:4 10.3389/fcimb.2012.00004 22919596 PMC3417403

[B15] Kim I. J. Blanke S. R. (2012). Remodeling the host environment: modulation of the gastric epithelium by the *Helicobacter pylori* vacuolating toxin (VacA). Front. Cell. Inf. Microbio. 2:37 10.3389/fcimb.2012.00037 22919629 PMC3417592

[B16] Liechti G. Goldberg J. B. (2012). Outer membrane biogenesis in *Escherichia coli*, *Neisseria meningitidis*, and *Helicobacter pylori*: paradigm deviations in *H. pylori*. Front. Cell. Inf. Microbio. 2:29 10.3389/fcimb.2012.00029 22919621 PMC3417575

[B17] Liu H. Semino-Mora C. Dubois A. (2012). Mechanism of *H. pylori* intracellular entry: an *in vitro* study. Front. Cell. Inf. Microbio. 2:13 10.3389/fcimb.2012.00013 22919605 PMC3417399

[B18] Matos R. G. Barria C. Pobre V. Andrade J. M. Arraiano C. M. (2012). Exoribonucleases as modulators of virulence in pathogenic bacteria. Front. Cell. Inf. Microbio. 2:65 10.3389/fcimb.2012.00065 22919656 PMC3417396

[B19] Miller W. G. Chapman M. H. Yee E. On S. L. McNulty D. K. Lastovica A. J. (2012). Multilocus sequence typing methods for the emerging campylobacter species, *C. hyointestinalis*, *C. lanienae*, *C. sputorum*, *C. concisus*, and *C. curvus*. Front. Cell. Inf. Microbio. 2:45 10.3389/fcimb.2012.00045 22919636 PMC3417633

[B20] Neal-McKinney J. M. Konkel M. E. (2012). The *Campylobacter jejuni* CiaC virulence protein is secreted from the flagellum and delivered to the cytosol of host cells. Front. Cell. Inf. Microbio. 2:31 10.3389/fcimb.2012.00031 22919623 PMC3417660

[B21] Noto J. M. Peek R. M. (2012). The role of microRNAs in *Helicobacter pylori* pathogenesis and gastric carcinogenesis. Front. Cell. Inf. Microbio. 1:21 10.3389/fcimb.2011.00021 22919587 PMC3417373

[B22] Croinin T. O. Backert S. (2012). Host epithelial cell invasion by *Campylobacter jejuni*: trigger or zipper mechanism? Front. Cell. Inf. Microbio. 2:25 10.3389/fcimb.2012.00025 22919617 PMC3417527

[B23] Pernitzsch S. R. Sharma C. M. (2012). Transcriptome complexity and riboregulation in the human pathogen *Helicobacter pylori*. Front. Cell. Inf. Microbio. 2:14 10.3389/fcimb.2012.00014 22919606 PMC3417511

[B24] Plummer P. J. (2012). LuxS and quorum-sensing in Campylobacter. Front. Cell. Inf. Microbio. 2:22 10.3389/fcimb.2012.00022 22919614 PMC3417632

[B25] Reid D. W. Muyskens J. B. Neal J. T. Gaddini G. W. Cho L. Y. Wandler A. M. (2012). Identification of genetic modifiers of CagA-induced epithelial disruption in Drosophila. Front. Cell. Inf. Microbio. 2:24 10.3389/fcimb.2012.00024 22919616 PMC3417398

[B26] Sorensen M. C. Van Alphen L. B. Fodor C. Crowley S. M. Christensen B. B. Szymanski C. M. (2012). Phase variable expression of capsular polysaccharide modifications allows *Campylobacter jejuni* to avoid bacteriophage infection in chickens. Front. Cell. Inf. Microbio. 2:11 10.3389/fcimb.2012.00011 22919603 PMC3417653

[B27] Stahl M. Butcher J. Stintzi A. (2012). Nutrient acquisition and metabolism by *Campylobacter jejuni*. Front. Cell. Inf. Microbio. 2:5 10.3389/fcimb.2012.00005 22919597 PMC3417520

[B28] Ta L. H. Hansen L. M. Sause W. E. Shiva O. Millstein A. Ottemann K. M. (2012). Conserved transcriptional unit organization of the cag pathogenicity island among *Helicobacter pylori* Strains. Front. Cell. Inf. Microbio. 2:46 10.3389/fcimb.2012.00046 22919637 PMC3417554

